# Anti-cancer immune priming with β-radioligand therapy using a novel high affinity antibody selectively targeting the 4Ig-Isoform of B7-H3

**DOI:** 10.7150/thno.123285

**Published:** 2026-03-12

**Authors:** Sarah E. Glazer, Margie N. Sutton, Ping Yang, Federica Pisaneschi, Manu Sebastian, Seth T. Gammon, David Piwnica-Worms

**Affiliations:** 1 Department of Cancer Systems Imaging, University of Texas MD Anderson Cancer Center, Houston, TX, USA.; 2 Department of Veterinary Medicine & Surgery, University of Texas MD Anderson Cancer Center, Houston, TX, USA.

## Abstract

**Rationale:**

Radioligand therapy (RLT) is an emerging oncologic strategy that uses molecularly targeted therapeutic radioisotopes to reduce tumor burden and improve survival in patients with advanced cancers. Expanding RLT to new targets and understanding its systemic immune effects could enhance its clinical impact. B7-H3 *(CD276)* is an attractive target due to its high differential expression in solid tumors compared to normal tissues. However, the presence of two isoforms, 4Ig-B7-H3 (tumor-associated) and 2Ig-B7-H3 (soluble decoy), poses challenges for selective targeting, especially in the context of RLT.

**Methods:**

A novel IgG2a monoclonal antibody (MIL33B) was developed with high affinity for 4Ig-B7-H3 (72 picomolar) and 8- to 18-fold selectivity over soluble 2Ig-B7-H3. Target specificity was assessed using live-cell fluorescence microscopy with AF594-labeled MIL33B. *In vivo* tumor binding was evaluated by PET-CT imaging with ^89^Zr-labeled MIL33B in murine xenograft (HeLa cervical) and syngeneic tumor models (4T1 breast, B16F10 melanoma, CT26 colorectal) expressing human 4Ig-B7-H3. Therapeutic efficacy was tested using ^90^Y-labeled MIL33B (100 μCi) in an established CT26 colorectal tumor model. Immunologic effects were analyzed to assess CD8+ T-cell activation and immune memory.

**Results:**

MIL33B demonstrated strong membranous localization using live cell fluorescence microscopy. PET-CT imaging with ^89^Zr-labeled MIL33B confirmed robust tumor-selective binding *in vivo*. A single systemic dose of ^90^Y-MIL33B achieved 53% long-term survival in a 4Ig-B7-H3-dependent manner in an otherwise fatal CT26 colorectal syngeneic tumor model. Immunologic analysis revealed that ^90^Y-MIL33B RLT acted as an immune priming event, engaging CD8+ T-cell activation and inducing immunological memory.

**Conclusions:**

MIL33B enables selective targeting of 4Ig-B7-H3 for beta-emitting RLT, overcoming challenges posed by soluble isoforms. These findings support further investigation of MIL33B as a systemic therapeutic with immune-priming potential, either alone or in combination strategies for cancer treatment.

## Introduction

**Figure 1:**
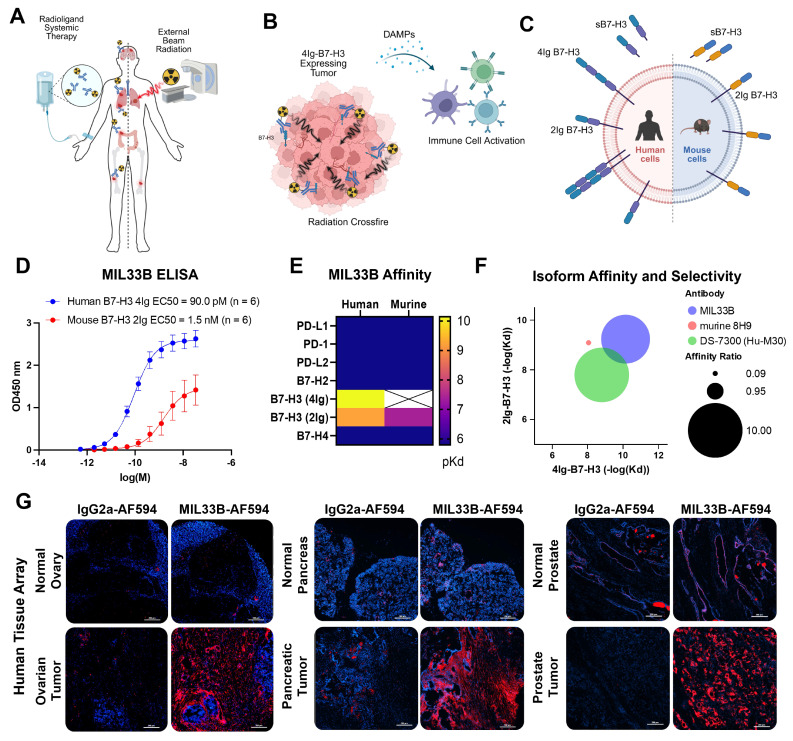
** Development of a high affinity, 4Ig-isoform selective antibody for beta-radioligand therapy. A)** Radioligand therapy (RLT) represents systemic therapy whereby the goal is to selectively bind and eliminate primary tumor sites as well as distant metastases and microscopic disease by delivering molecularly-targeted radioactivity through a single therapeutic intervention as compared to conventional external beam radiation therapy (EBRT) which targets with curative intent a defined radiation field localized to the primary tumor, and in some applications, to oligometastatic disease. **B)** Schematic describing the role of “Crossfire” whereby utilizing a 4Ig-B7-H3 molecularly targeted beta-radioligand therapy, the longer distance of the beta-particle deposition and the heterogenous expression of 4Ig-B7-H3 on the tumor and tumor microenvironment allow for a larger, yet still targeted deposition of radiation. Upon damage and tumor cell killing, damage-associated molecular patterns (DAMPs) may be released and provide immune priming signals. **C)** B7-H3 (*CD276*) is a type-I transmembrane immune modulatory protein with two isoforms found in humans (4Ig-B7-H3 and 2Ig-B7-H3) and a single isoform found in mice (2Ig-B7-H3). There is a soluble 2Ig-component that can be found in circulation, which may serve as a “sink” for non-differentiated isoform-specific anti-B7-H3 antibodies. **D)** ELISA of serial dilutions of MIL33B from different production lots demonstrated high affinity to plate-bound extracellular domain of human 4Ig-B7-H3 (EC50 = 90.0 pM, 95% CI: 62 pM - 130 pM; n = 6), and lower affinity to plate-bound extracellular domain of mouse 2Ig-B7-H3 (EC50 = 1.7nM, 95% CI: 0.47 nM - 5.0 nM; n = 6). **E)** Heatmap of biolayer interferometry analysis of MIL33B binding to B7-H3 homologues and common immune checkpoint proteins. MIL33B had the highest affinity for human 4Ig-B7-H3 and selection over the 2Ig-B7-H3 human isoform. **F)** MIL33B demonstrated greater isoform affinity and selectivity compared to other anti-B7-H3 antibodies. **G)** Immunofluorescence staining of fresh frozen human tissue microarrays with normal and paired tumor tissue. Images represent IgG2a-AF594 labeled isotype control (left, red) and MIL33B-AF594 (right, red) overlaid on DAPI (nuclear DNA, blue) for normal (top) and cancerous tissue (bottom). Scale bars represent 200 µm.

Radiotheranostics, also referred to as radioligand therapy (RLT), functions as a systemic modality designed to eradicate primary tumors, disseminated metastases, and occult micrometastatic disease through targeted delivery of cytotoxic radiation **(Figure 1A)** 1. By comparison, conventional external beam radiation therapy (EBRT) targets with curative intent a defined radiation field localized to the primary tumor, and in some applications, to oligometastatic disease 2, but many patients present with distant macro- and micro-metastases and continuously shed detectable levels of circulating tumor cells into the systemic circulation outside the radiation field 2. Both RLT and EBRT are conventionally thought to achieve their effect through direct radiation-induced toxicity to the tumor cell compartment via irreparable DNA damage; however, standard EBRT has been shown to evoke complex immune responses, with evidence for both immune activating (abscopal effect) and immune suppressive actions 2-4. Mechanistically, RLT induces tumor cell death via localized generation of radiation-induced reactive oxygen species (ROS), single/double DNA strand breaks, and oxidative stress, with collateral effects on the TIME 1, 5-7. Despite clinical successes, the narrow spectrum of FDA-approved targets and the opportunity for enhanced understanding of mechanisms of action of RLT portend an unmet medical need to expand the repertoire of second-generation targets and high-affinity ligands.

In this regard, B7-H3 (*CD276*), a homologue of the B7 superfamily of cell-surface immune co-regulatory proteins, is found in a wide variety of cancers (colon, renal, cervical, esophageal, hepatocellular, neuroblastoma, breast, pancreatic, prostate, head and neck, glioblastoma, ovarian, and NSCLC, among others) 8-16, correlating with poor prognosis, reduced survival, and increased metastasis 17. While *B7-H3* mRNA is readily detected in most human tissues, by contrast, B7-H3 protein expression is tightly regulated by microRNAs, particularly miR-29, leading to significant differential protein expression between tumors and normal tissues 18. B7-H3 protein is present in only a subset of normal cells and tissues, such as placenta and testis, wherein the extracellular domain of B7-H3 would be poorly accessible to a targeting agent in the blood 19, 20. Compared to other radioligand therapy targets, such as PSMA or HER2, which exhibit antigen densities of ~5x10^5^ to 10^6^ molecules/cell 21, 22, B7-H3 expression on solid tumors is widespread, but more heterogeneous. Some tumor types, such as breast and lung cancers, show modest expression (~10^4^ to 5x10^5^ molecules/cell) 23, while others, such as neuroblastoma and prostate cancer 24, 25, show high level expression of B7-H3. Furthermore, B7-H3 protein can be induced on multiple components of the TIME (cancer cells, cancer-associated vasculature, neutrophils, monocytes, dendritic cells and other antigen-presenting cells 8,26-33), and thus, the combination of tumor/tumor-associated selective expression with minimal normal tissue expression portend a favorable therapeutic index, attracting interest as an oncologic target, including for RLT 8, 33-36** (Figure 1B)**.

Importantly, human B7-H3 is a type 1 transmembrane protein expressed in two isoforms, a 4Ig-B7-H3 isoform consisting of an extracellular ectodomain comprising two pairs of immunoglobulin constant and variable domains (IgC/IgV, IgC/IgV) 26, 37-39, and a 2Ig-B7-H3 isoform containing only one extracellular IgC/IgV pair **(Figure 1C)** 40. This stands in contrast to rodents, wherein only the 2Ig isoform is expressed 39, and may be responsible for the species-specific differential roles of B7-H3 in regulating immune checkpoint observed in early studies 41. In humans, a soluble component (s2Ig-B7-H3), likely representing ectodomain shedding of B7-H3, similar to other type 1 and 2 membrane proteins and members of the B7 family 42, 43, has been identified and accumulates in compartments outside the TIME, such as the blood pool and cerebral spinal fluid 44-46. Increased circulating s2Ig-B7-H3 has been identified in the serum of patients during progression of malignancies and inflammatory states 47-51. This is of particular concern in the context of imaging agent and low-mass RLT therapeutic development because s2Ig-B7-H3 can act as a decoy target and ligand sink, potentially diminishing efficacy and increasing toxicities of ligands that target both isoforms. None-the-less, abundant evidence indicates that 4Ig-B7-H3 is the dominant isoform present on the cell surface of human cancers 26, 37-39, 52, and unique to B7 family members, the presence of 4Ig and 2Ig isoforms may provide a “handle” to develop a 4Ig-tumor-selective ligand, such as an antibody, for RLT to overcome challenges presented by circulating B7 isoforms.

Herein, a novel targeted beta-radioligand antibody therapeutic with 8- to 18-fold selectivity for the 4Ig isoform of B7-H3 compared to the 2Ig isoform was extensively validated *in vitro* and *in vivo,* yielding functional cures in syngeneic immunocompetent murine tumor models. The biochemical B7-H3 targeting mechanism was firmly established, and the high cure rate in a syngeneic model opened the opportunity to study systemic immune responses and long-term anti-tumor memory. Mechanistically, 4Ig-B7-H3-targeted beta-RLT functioned as a primary immune priming event shown to promote downstream CD8^+^ T-cell activation and induce immunological memory* in vivo*, thus experimentally reinforcing the concept that targeted beta-RLT ultimately engages systemic adaptive immunity.

## Materials & Brief Methods

See **Supplementary Materials** for detailed methods.

### Antibody Development and MIL33B Production

The MDACC Monoclonal Antibody Core Facility (protocol 00000620-RN01 through RN03) was utilized to develop anti-B7-H3 monoclonal antibodies. Briefly, mouse L cells transfected using polybrene with a lentivirus containing the *homo sapiens CD276* transcript variant 1 mRNA (NM_001024736.1) (Genecopia LPP-Z3060-LV105-100) and puromycin suppression was used for selection. New Zealand White mice (NZBWF1/J, Jackson Laboratory) or Balb/c (Charles River) were immunized with the cells by foot-pad immunization 133. Sera obtained from immunized mice were screened for differential binding to human 4Ig-B7-H3-expressing or vector control transcript (Genecopia LPP-NEG-Lv105-025-c) and parental cells as well as binding to mouse 2Ig-B7-H3 protein (R&D 1397-B3-050) by ELISA. Selected clones were used to generate stable hybridomas. Monoclonal antibodies obtained from the supernatants of these hybridomas were further evaluated for human, mouse and porcine 4Ig-/2Ig-B7-H3 affinity and specificity.

MIL33B was purified from supernatants from the MIL33B hybridoma by either the MDACC Monoclonal Antibody Core or by BioXCell. ELISA was used to validate the affinity of each MIL33B stock for human and mouse 4Ig-/2Ig-B7-H3.

### ELISA and Bi-Layer Interferometry Analysis

ELISA was performed using MIL33B to probe plate-bound human and mouse B7-H3 extracellular domains (human 4Ig-B7-H3 protein R&D 2318-B3-050; mouse 2Ig-B7-H3 protein R&D 1397-B3-050), with 1:2000 dilutions of secondary antibody, (goat anti-mouse IgG (Fc) HRP (Biorad)). OCTET RED384 platform, a technology based on Bio-Layer Interferometry (BLI) was utilized to further characterize antibodies and their binding kinetics. Briefly, the murine-derived anti-B7-H3 clone, MIL33B, was immobilized on AMC biosensors using anti-mouse IgG Fc capture at a fixed concentration of 10 μg/ml. Loaded biosensors were subsequently interacted with 7 serial dilutions (ranging from 3 nM to 200 nM) of the extracellular domain of various B7-H3 isoforms or relevant B7 protein homologues. Association and dissociation constants, Ka and Kd, respectively, and calculated affinity constants (K_D_) were measured using Octet Analysis Studio 13.

### Human Tissue Microarray Fluorescence Staining

Frozen human tissue microarrays were purchased from Fisher Scientific (50-180-886) containing arrayed normal and cancer tissues for multiple organ sites. Staining was performed following a brief fix (5 minutes) at room temperature of the slide in 4% paraformaldehyde. Following fixation, slides were washed two times in 5%BSA/PBS for 5 minutes each. Blocking was performed in 5% BSA/PBS containing 1:100 IgG2a (Final concentration~ 50 µg/mL) at room temperature for 30 minutes. Primary antibody was added in 1% BSA/PBS and incubated overnight at 4ºC protected from light. MIL33B-AF594 and IgG2a-AF594 were diluted 1:100; final concentration was 10 µg/mL. Following overnight incubation, slides were washed 2X in 1%BSA/PBS for 5 minutes each. Nuclear staining was performed with DAPI at a dilution of 1:1000 in 1%BSA/PBS for 10 minutes at room temperature. Slides were washed an additional time in 1%BSA/PBS and mounted using antifade mounting media. Once dried, the slides were sealed and images were captured on a Nikon TiE Epifluorescence microscope.

### Cell Line Generation

B16F10 murine melanoma cells, 4T1 murine triple negative breast cancer cells, and CT26 murine colorectal carcinoma cells were transfected using polybrene with lentiviral constructs containing the *homo sapien CD276* transcript variant 1 mRNA (NM_001024736.1) (Genecopia LPP-Z3060-LV105-100) or the negative vector control transcript (Genecopia LPP-NEG-Lv105-025-c) and selected in puromycin. CD276^-/-^ cells were generated by transfecting HeLa parental cells with human-derived *CD276* CRISPR/Cas9 pooled plasmids (Santa Cruz Biotechnology sc-402032) or double nickase control plasmids (each containing a GFP marker, three separate guide RNAs, and a plasmid encoding Cas9). GPF-positive cells were sorted and expanded. HCT116 cells or HCT116 cells stably transfected with a *KB5-IkBα-FLUC* reporter 134 were cultured as described.

### Live Cell Fluorescence Microscopy

Cells were plated in a 4-well Ibidi dish at 5x10^4^ cells in 500 µL of media. Cells were incubated at 37 ºC in 5% CO_2_ for 24-48 hours. MIL33B or mouse isotype control IgG2a (BioXcell BE0085) were labeled with Alexa594 (Thermofisher A20185) by the MDACC flow cytometry core. Subsequently, cells were incubated with 10 µg/mL of either Alexa549-labeled MIL33B or IgG2a and 5 µL of a 1:100 dilution in PBS of Hoechst stain for 1 hour and imaged on a Nikon TiE inverted microscope (40x objective).

### Antibody Conjugation with p-SCN-Bn-Deferoxamine or p-SCN-Bn-DOTA

For antibodies conjugated with p-SCN-Bn-Deferoxamine (Macrocyclics), 4-7 mg/mL of either MIL33B or mouse IgG2a (BioXcell) were desalted into 0.1M NaHCO_3_ (pH = 8.4) using 7K MWCO Zeba (Thermofisher) protein desalting spin columns. When conjugating MIL33B to p-SCN-Bn-DOTA (Macrocyclics), the stock solution was concentrated to 9-11 mg /mL using Amicon Ultra-0.5 centrifugal filter units with a 30 kDa cutoff, and then similarly desalted into a 0.1 M solution of sodium bicarbonate (pH = 8.4). 5 mg of each respective chelator dissolved in DMSO were incubated with the respective antibodies for 1 hour at 37ºC with periodic mixing. Final antibodies were desalted into 0.1 M ammonium acetate (pH = 6.6-7) as described previously 135.

### Radiolabeling of MIL33B with Zirconium-89

The MDACC cyclotron facility or the University of Wisconsin cyclotron facility produced Zirconium-89 in 1 M oxalic acid. Zirconium-89 oxalate (3 mCi) was neutralized to pH 7 by 1.0 M Na_2_CO_3_ (pH = 11). Following, 20-30 µL of a 4-7 mg/mL solution of DFO-conjugated antibodies, MIL33B or mouse IgG2a, was added to the buffer and the final volume brought to 200 µL with PBS and with zirconium-89 oxalate for 1 hour at 37 ºC. Chelation efficiency was evaluated by radio-TLC with antibody solutions quenched in 50 mM DPTA (pH = 7) using 50 mM DPTA (pH = 7) as the running solvent. A PD-10 column was used to purify chelated antibodies into PBS. Radio-TLC with antibody solutions quenched in 50 mM DPTA pH 7 and with 50 mM DPTA pH 7 as the running solvent was used to determine chelation efficiency. Purity was validated by radio-TLC and radio-SEC-HPLC with a 200 10-30 Superdex column (71 minute isocratic elution in PBS with 0.1 M NaCl and 0.05% sodium azide). ^89^Zr-labeled antibodies were assessed for stability at room temperature in PBS and evaluated at 4 hours, 24 hours and 72 hours after radiolabeling by radio-SEC-HPLC.

### Radiolabeling of MIL33B with Yttrium-90

[^90^Y]YCl_3_ in 0.04 M HCl was purchased from Eckert & Ziegler Radiopharma. [^90^Y]Yttrium chloride was buffered in an equal volume of 0.1 M ammonium acetate (pH = 5.6). Following, 50 µL of DOTA-MIL33B was added to the solution. DOTA-MIL33B was incubated with [^90^Y]yttrium chloride for 1 hour at 37ºC. Chelation efficiency was evaluated by radio-TLC with antibody solutions quenched in 50 mM EDTA using 10 mM EDTA as the running solvent. ^90^Y-DOTA-MIL33B were purified into PBS using a PD-10 column. Purity was validated by radio-TLC and radio-SEC-HPLC with a 200 10-30 Superdex column (71 minute isocratic elution in PBS with 0.1 M NaCl and 0.05% sodium azide). The stability of ^90^Y-DOTA-ML33B incubated at room temperature in PBS was evaluated at 4 hours, 24 hours and 48 hours after radiolabeling by radio-SEC-HPLC.

### Tumor Models

All experiments were approved under an MDACC IACUC protocol (00001179-RN01 and RN02). Mice aged 5-12 weeks were used for all experiments. Tumor cells were implanted subcutaneously on the flanks of female athymic nude immunodeficient mice strain 490 (Charles River) or flanks of respective immunocompetent female Balb/c mice (Taconic) or female C57BL/6 mice (Taconic). For all mice, tumors were measured using calipers twice weekly. Mice were defined to reach endpoint when either the length or width of the tumor reached 15 mm or the tumors had ulcers of 3 mm, at which point mice were euthanized by CO_2_ asphyxiation and cervical dislocation.

### PET-CT Imaging Methods and Analysis

Mice were injected with approximately 20-60 μCi of either ^89^Zr-DFO-MIL33B or ^89^Zr-DFO-IgG2a intravenously. For cold blocking experiments, mice received 200 µg of un-labeled MIL33B 1 hour before injection with the tracer of interest. Mice were imaged at 24 hours, 72 hours and 144 hours on a PET-CT scanner (Albira, Bruker), with a field of view of 120 mm for PET and 70-75 mm for CT. Mice were imaged with a single 10 minute PET scan and a fast CT HV, HD scan. VOIs were drawn in PMOD and used to calculate %ID/cc for respective VOIs at each time point.

### Cherenkov Radiation Optical Imaging

Images of mice were obtained using an IVIS Spectrum, open filter, FOV 23 cm x 23 cm, and image acquisition time of 5 minutes, followed by acquisition of a bright light image.

### ^90^Y-DOTA-MIL33B Treatment Strategy

Mice harboring CT26 4Ig-B7-H3 or neg vector tumors were selected for tumors with a volume of about 50 mm^3^ - 150 mm^3^ at 10-12 days post tumor implantation and randomized into treatment groups. Untreated mice were followed regardless of initial tumor size. Mice were treated with intravenous injection of 100 µCi of ^90^Y-DOTA-MIL33B or 100 µL of sterile saline. For HeLa B7-H3^+/+^ WT or HeLa B7-H3^-/-^ KO, 5x10^6^ cells were implanted subcutaneously in the flanks of athymic nude mice. Mice harboring HeLa B7-H3^+/+^ or HeLa B7-H3^-/-^ KO tumors were not size selected and received an intravenous injection of 100 µCi of ^90^Y-DOTA-MIL33B 30 days post tumor implantation or were not treated (serving as a tumor growth rate control), thus anchoring survival with previously performed animal studies 59.

### Tumor Growth Rate Comparison

In comparing the growth rates of HeLa B7-H3^+/+^ WT or HeLa B7-H3^-/-^ KO tumors treated with ^90^Y-DOTA-MIL33B i.v., changes in tumor growth were calculated on a mouse by mouse basis by calculating the change in tumor size at each time point compared to initial tumor size **(Figure S10)**. Slopes of tumor growth (growth rates) were calculated with a 3-point exponential fitting curve using Prism software.

### Immune Cell Depletion *In Vivo*

Mice harboring CT26 4Ig-B7-H3 tumors were treated with a single dose of 100 µCi of ^90^Y-DOTA-MIL33B i.v.; 1 day before treatment, mice received either 250 µg of anti-mouse CD8b (clone 53-5.8 BioXcell), anti-mouse CD4 (clone GK1.5 BioXcell), or anti-rat IgG polyclonal (BioXcell) i.p. and then received subsequent doses of each respective depleting antibody 2 days, 6 days and 9 days post treatment with ^90^Y-DOTA-MIL33B i.v. Response to treatment with depleting antibodies was monitored by caliper measurements of the tumors.

### Tumor Cell Re-Challenge

Mice harboring CT26 4Ig-B7-H3 tumors that had previously been treated with 100 µCi of ^90^Y-DOTA-MIL33B were followed for 100 days post tumor implantation. Mice whose tumors had regressed and did not recur at the 100-day time point were deemed long-term survivors. Long-term survivors were implanted with 5x10^4^ CT26 4Ig-B7-H3 or CT26 neg vector control cells in 100 µL of a 1:1 ratio of PBS:matrigel subcutaneously on the left flank. Age-matched naïve female Balb/c mice (Taconic Biosciences) were also implanted identically with 5x10^4^ CT26 4Ig-B7-H3 or CT26 neg vector control cells in 100 µL of a 1:1 ratio of PBS:matrigel subcutaneously on the left flank. Response to the tumor cell re-challenge was monitored by caliper measurements.

### MIL33B Monotherapy

Balb/c female mice (Taconic) were implanted with 1x10^5^ CT26 4Ig-B7-H3 cells in 100 µL PBS subcutaneously in the right flank. Mice received 200 µg i.p. of either MIL33B, mouse IgG2a isotype control antibody (Bioxcell), or 100 µL PBS on day 3, 6, 9, 12, 15, 18, 21, and 24 post cell implantation.

### Histology

Mice harboring CT26 4Ig-B7-H3 tumors were treated with 100 µCi of ^90^Y-DOTA-MIL33B i.v. according to the protocol. 6 days after treatment, mice were sacrificed, tumors fixed in formalin for 24-36 hours and then subsequently stored in 70% ethanol for radioactive decay. Tumors were stained by H&E staining, anti-CD4 (Abcam ab183685), anti-CD3 (Cell Signaling 99940), anti-CD8 (Abcam ab217344), anti-Ki67 (Cell Signaling 12202), anti-CD31 (Abcam ab124432) and anti-cleaved caspase-3 (Cell Signaling 9662) by the MDACC histology core facility. Histopathology review, including immuno-histochemistry scoring, was performed by a board-certified pathologist.

### Multiplexed Immunofluorescence Staining

Multiplexed Immunofluorescence (mIF) staining was performed on tissues collected and processed as previously described (Histology section). Using the established Lunaphore COMET system, FFPE tissues were placed in tanks containing BioGenex EZ Elegans AR2 buffer, enclosed inside a BioGenex EZ Retriever microwave system. The samples were heated in the microwave to 107°C for 15 minutes to dewax, rehydrate and retrieve the antigens. After heating, the slides were cooled to room temperature and loaded into the COMET system where they were covered and sealed by a fast-fluidic exchange microfluidics chip for staining and imaging in the instrument. Cycle temperature remained at 37°C for the duration of the staining, incubation time was 16 minutes for primary and 4 minutes for secondary antibodies, and image capture was obtained at exposure times of 25 ms, 250 ms or 400 ms. The following antibodies were used at the given dilutions below: Cycle 1: CD8 (Bethyl, BLR173J, Channel: Cy5, Dilution: 1:100) Cycle 2: CD4 (CST, D7D27, Chanel: Tritc, Dilution1:100); Cycle 3: TCF1/TCF7 (CST, C63D9, Channel: Cy5, Dilution: 1:100); Cycle 4: B7-H3 (R&D, AF1397, Channel: Cy5, Dilution: 1:200). COMET collected images in OME-Tiff formats, which were scanned and visualized in Comet Viewer (Lunaphore). Image analysis was performed using Oncotopix Discovery (Visiopharm, v.2023.01).

### RNA sequencing and Single Cell RNA-Seq

CT26 4Ig-B7-H3 and CT26 negative vector cells were seeded at 70% confluency for 24 hours before RNA isolation was performed using QIAGEN RNA isolation kit (Kit #74104). Purified RNA underwent quality control and then the Poly(A) RNA sequencing library was prepared following Illumina's TruSeq-stranded-mRNA sample preparation protocol. RNA integrity was checked with Agilent Technologies 2100 Bioanalyzer. Poly(A) tail-containing mRNAs were purified using oligo-(dT) magnetic beads with two rounds of purification. After purification, poly(A) RNA was fragmented using divalent cation buffer at elevated temperature. Quality control analysis and quantification of the sequencing library were performed using Agilent Technologies 2100 Bioanalyzer High Sensitivity DNA Chip. Paired-ended sequencing was performed on Illumina's NovaSeq 6000 sequencing system (LC Sciences). Data analysis was performed by LC Sciences using Cutadapt and perl scripts to remove the reads that contained adaptor contamination, low quality bases, and undetermined bases. Sequence quality was verified using FastQC and map reads to the genome were performed using HISAT2. To assemble the reads, StringTie was used. Perl scripts and gffcompare were utilized to merge all transcriptomes and reconstruct a comprehensive transcriptome. StringTie and ballgown were used to estimate the expression level of all transcripts.

C57Bl/6 mice (Taconic) at 10-11 weeks old received subcutaneous injections of 5x10^4^ cells on the right flank with either B16F10 4Ig-B7-H3 cells or B16F10 neg vector cells. Tumors were extracted 38 days after implantation. Cells were digested into a single cell suspension using previously published protocols 136. Individual cells were captured using the 10x Genomics Chromium Single Cell 3′ Library per the manufacturers' protocol and a subsequent cDNA library was generated and sequenced on a NovaSeq6000 S2 sequencer. Data were initially processed and quality controlled using the Cell Ranger Software Pipeline. Cell types were classified based on previously defined cell markers 137. Data were clustered and dimensionally reduced using UMAP (Uniform Manifold Approximation Projection) utilizing the Seurat R Package 138, 139.

### Statistics

Statistics were calculated using GraphPad Prism 9.0.0 (121). Data are represented as mean ± standard error. Pairs were compared using a student's t-test where p-values less than or equal to 0.05 were considered significant.

## Results and Discussion

### Development of a High Affinity, 4Ig-Isoform Selective Antibody for Beta-Radioligand Therapy

To develop a 4Ig-B7-H3-specific antibody with both high affinity to the folded extracellular domain of the human 4Ig isoform and selectivity over the s2Ig isoform, New Zealand White (NZBWF1/J) or Balb/C mice were immunized by foot pad injection with murine L-cells transduced with cassettes expressing human 4Ig-B7-H3 on the cell surface. Host sera were screened by ELISA to assess binding to the extracellular domains of both human 4Ig-B7-H3 and murine 2Ig-B7-H3, and candidate hybridomas subsequently scaled for further evaluation. Counter-screening was performed for all candidates utilizing live cell fluorescence binding assays to HeLa cells, which are known to express 4Ig-B7-H3 and secrete s2Ig-B7-H3 53, yielding 3 preliminary antibody candidates that could bind to folded, glycosylated human 4Ig-B7-H3 in the face of s2Ig-B7-H3 *in cellulo*. Final prioritization of leads was ultimately based on high affinity binding by ELISA to folded human 4Ig-B7-H3 **(Figure 1E)**. Subsequently, hybridoma expansion was performed to further evaluate murine monoclonal antibody candidate 33B, hereafter known as Molecular Imaging Laboratory 33B (MIL33B), isotype IgG2a, for affinity and specificity against various B7 family members, animal species and isoforms.

**Figure 2:**
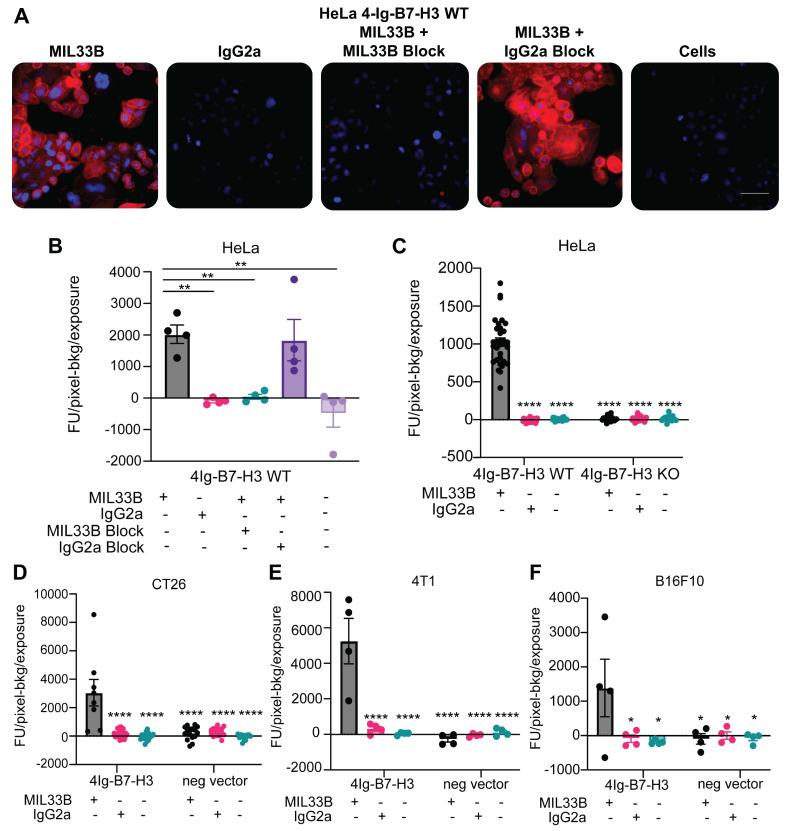
** MIL33B has high affinity and specificity for the human 4Ig-B7-H3 isoform. A. Immunofluorescence live-cell staining of multiple 4Ig-B7-H3 expressing cell lines demonstrated 4Ig-B7-H3 specific binding and membrane localization of MIL33B.** High endogenous 4Ig-B7-H3-expressing human HeLa cells incubated with Alexa549-labeled MIL33B demonstrated high intensity membrane-specific localization compared to Alexa549-labeled IgG2a or cells alone when incubated for 1 h. Pre-incubation with un-labeled MIL33B was superior to murine IgG2a in displacing Alexa549-labeled MIL33B (cold block). Scale bar = 100 µM **B-F.** Quantification of MIL33B-AF594 or IgG2a-AF594 staining intensity (Fluorescence Units-Background/Seconds of the entire field) following 1 hour incubation with antibodies at 37ºC and 5% CO_2_ humidity. HeLa WT **(B)**, HeLa WT and KO **(C)**, CT26 **(D)**, 4T1 **(E)**, or B16F10 **(F)** were cultured under normal conditions.

Affinity to the folded ectodomain of human 4Ig-B7-H3 in an ELISA format yielded an EC_50_ of 90.0 pM (95% CI: 62 pM - 130 pM; n = 6), substantially higher affinity (18-fold) compared to the plate-bound extracellular domain of murine 2Ig-B7-H3 (EC_50_ = 1.7 nM; 95% CI: 0.47 nM - 5.0 nM; n = 6) **(Figure 1D, Figure 1S)**. Next, MIL33B binding kinetics were measured for folded human, murine and porcine B7-H3 isoforms by biolayer interferometry as a model of binding selectivity between the 4Ig extracellular domain and s2Ig in circulation, resulting in calculated K_D_ values of 7.23x10^-11^ M for human 4Ig-B7-H3, 5.80x10^-10^ M for human 2Ig-B7-H3, 4.11x10^-8^ M for mouse 2Ig-B7-H3, and 1.02x10^-10^ M for porcine 4Ig-B7-H3 **(Table 1, Figure 1E, Figure 2S)**. These profiles for MIL33B demonstrated significantly higher affinity for 4Ig-B7-H3 when compared to other B7-H3 antibodies that have been tested clinically, e.g., 8H9 and MGA271 54-58. The 8-fold selectivity for the human 4Ig isoform compared to the human 2Ig isoform **(Table 1, Figure 1F)** presented MIL33B the opportunity to enhance tumor-targeting by traversing soluble decoy in circulation.

**Figure 3:**
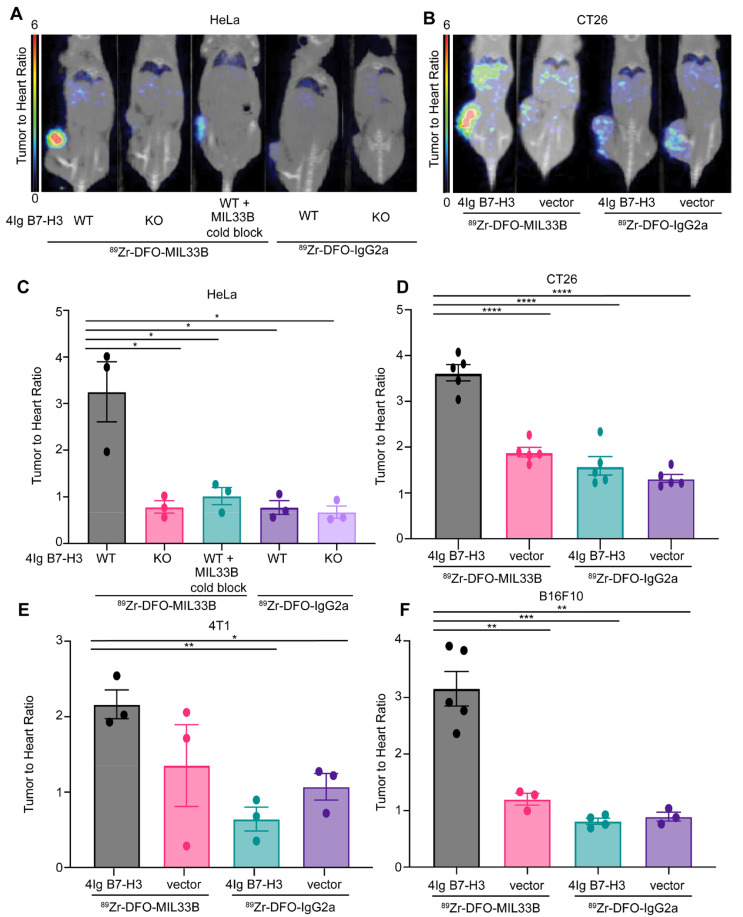
** Immuno-PET demonstrates MIL33B targeting to tumor-specific 4Ig-B7-H3 *in vivo*.** PET/CT images, SUV normalized to heart to correct for animal-to-animal variability in clearance between genotypes for HeLa xenografts (A) and CT26 syngeneic tumor models (B). Images acquired at 72 hours post-injection revealed significantly higher binding and retention of ^89^Zr-DFO-MIL33B in mice harboring HeLa B7-H3 +/+ tumors compared to HeLa B7-H3-/- tumors or HeLa B7-H3+/+ tumors pre-treated with cold MIL33B, or HeLa B7-H3+/+ tumors and HeLa B7-H3-/- tumors imaged with ^89^Zr-DFO-IgG2a (A). Similar on-target (B7-H3 expressing tumor) uptake was observed using the syngeneic mouse model for colorectal cancer, CT26 (B). Normalized SUV of ^89^Zr-DFO-MIL33B was significantly retained within 4Ig-B7-H3 expressing tumors compared to genotype negative controls or using isotype control, ^89^Zr-DFO-IgG2a. The phenotype was observed in four different models including the human cervical cancer cell line, HeLa (C), as well as several murine syngeneic models transduced with human 4Ig-B7-H3 compared to respective tumors transfected with negative vector control or imaged with ^89^Zr-DFO-IgG2a; CT26 (D), triple negative breast cancer model 4T1 (E), melanoma model B16F10 (F). Comparisons are determined by un-paired two tailed t-tests, *p < 0.05, **p < 0.01, ***p < 0.001.

The specificity of MIL33B for B7-H3 versus B7 family members was further evaluated by determination of the affinity of MIL33B to other human and mouse B7 family members and common immune checkpoint proteins. K_D_ values by ELISA of MIL33B against plate-bound mouse and human B7-H2, B7-H4, PD-L1, PD-L2, PD-1, and CTLA-4 were not measurable (>1x10^-6^ M) **(Figure 1E)**, yielding > 5 orders of magnitude selectivity. Similarly, biolayer interferometry analysis of the binding of MIL33B to human and mouse B7-H2, B7-H4, B7-H1(PD-L1), PD-L2, and PD-1 were also below the limit of detection demonstrating orders of magnitude of selectivity **(Table 2 and Figure 3S)**.

### Validation of Selective Binding of CDRs of MIL33B to Human Cancers versus Normal Tissues

To evaluate tumor-differentiated binding of B7-H3 by MIL33B, fixed-frozen human tissue arrays containing microarrayed tissue from multiple organ sites of “normal” or “tumor” tissue were stained using MIL33B labeled with AF594 or the appropriate IgG2a-AF594 labeled control antibody. Differential and tumor-specific binding was observed using MIL33B-AF594 for several tumor types including ovarian, pancreatic, and prostate tumors **(Figure 1G)**.

### Validation of CDR Binding and 4Ig-B7-H3 Protein Targeting by Live Cell Fluorescence Microscopy

**Figure 4:**
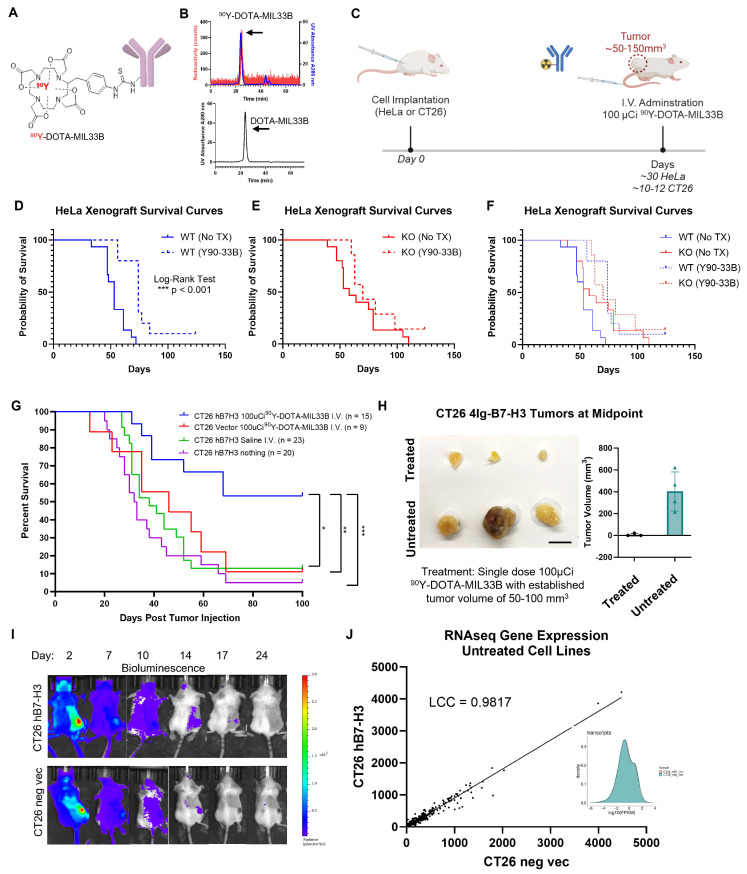
** Therapeutic efficacy of I.V. ^90^Y-DOTA-MIL33B *in vivo*. A.** Diagram of ^90^Y-DOTA-MIL33B. **B.** Radio-HPLC was used to document the labeling efficiency of ^90^Y-DOTA-MIL33B. **C.** Experimental schema used for *in vivo* studies to deliver a single dose of 100 μCi ^90^Y-DOTA-MIL33B or saline for tumor treatment. **D.** 5 x 10^6^ HeLa WT cells were injected into the right flank of athymic nude mice and allowed to establish a tumor for 30 days prior to treatment of half of the mice with ^90^Y-DOTA-MIL33B. Survival curves for untreated (solid blue) or treated (dashed blue) were plotted. Single dose treatment with ^90^Y-DOTA-MIL33B significantly extended survival *** p < 0.001. **E.** 5 x 10^6^ HeLa KO cells were injected into the right flank of athymic nude mice and allowed to establish a tumor for 30 days prior to treatment of half of the mice with ^90^Y-DOTA-MIL33B. Survival curves for untreated (solid red) or treated (dashed red) were plotted. **F.** Kaplan-Meier survival curves for WT and KO HeLa xenografts were plotted demonstrating the right shift in survival following knockout of B7-H3 from the tumor compartment, and further right shifting with anti-B7-H3 targeted radioligand therapy, ^90^Y-DOTA-MIL33B. **G.** CT26 (vector control or h(4Ig)B7-H3 transduced) tumors were implanted subcutaneously in BALB/c mice and allowed to establish a size between 50-100 mm^3^ (10-12 days) prior to treatment with saline or a single dose of ^90^Y-DOTA-MIL33B (100 μCi). Kaplan-Meier survival curves were plotted for CT26-hB7-H3 tumors untreated (purple), CT26-hB7-H3 tumors treated with i.v. saline (green), CT26-vector tumors treated with ^90^Y-DOTA-MIL33B (100 μCi) (red), and CT26-hB7-H3 tumors treated with ^90^Y-DOTA-MIL33B (100 μCi) (blue). 4Ig-B7-H3 expressing CT26 tumors treated with a single dose of ^90^Y-DOTA-MIL33B (100 μCi) had significantly prolonged survival compared to all other treatment groups. **H.** Midpoint tumors collected 7 days post treatment with 100 μCi ^90^Y-DOTA-MIL33B for three mice in each group were excised and photographed. Tumor volume for all midpoint tumors was plotted for CT26-hB7-H3 that were treated vs. untreated (N=4 in each group). Scale bar = 1 cm. **I.** Cherenkov imaging of ^90^Y-DOTA-MIL33B. Mice harboring CT26 hB7-H3 tumors and CT26-vector tumors were imaged on an IVIS Spectrum 2, 7, 10, 14, 17 and 24 days after I.V. administration of 100 µCi ^90^Y-DOTA-MIL33B. Measurement of Cherenkov radiation demonstrated tumor uptake greater in CT26-hB7-H3 than CT26-vector tumors, that diminished overtime. **J.** RNA-sequencing of CT26 4Ig-B7-H3 and CT26 empty vector control cells were performed and gene expression profiles between the cell lines were compared. Gene expression profiles revealed a Lin's Concordance Correlation Coefficient of 0.9817. Density of gene/transcript expression for the two cell lines is presented as density by log10(FPKM) (inset).

To evaluate B7-H3 targeting of MIL33B *in cellulo* (and later* in vivo*), several high and low 4Ig-B7-H3-expressing human and mouse cancer cell lines were generated. By Western blot analysis, parental HeLa cells (HeLa^+/+^) showed relatively high endogenous expression of human 4Ig-B7-H3 and low 2Ig-B7-H3 **(Figure 4SA)**, while a clonal HeLa B7-H3 CRISPR/Cas9 knockout cell line (HeLa^-/-^; KO) showed no detectable protein 59. As expected, 4T1 (murine triple negative breast cancer), B16F10 (murine melanoma cell lines), and CT26 (murine colon cancer) demonstrated minimal expression of the murine 2Ig-B7-H3 isoform relative to human tumor cells **(Figure 4SA)**. Note that the commercial antibody leveraged in the Western blot experiments likely overestimated the levels of rodent versus human protein as its primary cognate antigen was mouse 2Ig with cross-reactivity to the human protein. To develop murine lines containing the human 4Ig-isoform of B7-H3, 4T1, B16F10, and CT26 cells were batch transfected with human 4Ig-B7-H3 lentiviral particles, generating cells expressing human 4Ig-B7-H3 protein at levels comparable or less than to those observed in human cancer cells **(Figure 4SA)**.

Next, MIL33B binding and specificity for human 4Ig-B7-H3 were tested *in cellulo*. MIL33B and a matched non-targeted isotype control antibody (IgG2a) were labeled with Alexa594. HeLa 4Ig-B7-H3^+/+^, HeLa B7-H3^-/-^, 4T1 4Ig-B7-H3, 4T1 negative vector, B16F10 4Ig-B7-H3, B16F10 negative vector, CT26 4Ig-B7-H3, and CT26 negative vector cells were incubated with either MIL33B-Alexa549, mouse IgG2a-Alexa549, or no antibody. Cell-associated antibody was assessed by live cell fluorescence microscopy, which enabled evaluation of both membrane association and internalization of antibodies over time. MIL33B demonstrated high cell-specific binding to endogenously high 4Ig-B7-H3-expressing human cells (HeLa^+/+^) and cells transduced with human 4Ig-B7-H3 (CT26 4Ig-B7-H3, 4T1 4Ig-B7-H3 and B16F10 4Ig-B7-H3) with a prominent plasma membrane pattern and evidence of internalization by 40 min to 1 h. MIL33B selectivity for 4Ig-B7-H3 versus other tumor surface antigens, FcR binding, and macropinocytosis was demonstrated via the vastly superior binding to cells expressing 4Ig-B7-H3 versus various negative controls, including B7-H3 Hela KO cells, negative vector control murine cells (reflecting low endogenous murine 2Ig-B7-H3), cells incubated with mouse IgG2a-Alexa549, and autofluorescence of unlabeled cells alone **(Figure 2A-F, Figure 4SB-C)**. Of note, we observed heterogeneous 4Ig-B7-H3-specific cell labeling of MIL33B in murine cell lines transduced with human 4Ig-B7-H3, consistent with expectations for cells not clonally selected for high 4Ig-B7-H3 expression after transfection. Further specificity of MIL33B versus non-specific membrane adhesion and macropinocytosis was independently validated by pre-incubating HeLa cells with excess un-labeled MIL33B, which blocked MIL33B-Alexa549 binding as accessed by live cell fluorescence microscopy **(Figure 2B)**. In addition, because the MIL33B isotype was mouse IgG2a, which has high affinity to mouse FcγR 60, additional validation of specificity versus FcγR binding was evaluated. Indeed, MIL33B pre-blocking was far superior to IgG2a pre-blocking, indicting no substantial Fc-mediated binding to solid tumor-expressed Fc receptors, consistent with the previously demonstrated superiority of MIL33B-AF594 compared to murine IgG2a-AF594 live cell binding and retention **(Figure 1C)**. Overall, MIL33B showed high specificity for human 4Ig-B7-H3 protein versus other tumor antigens, tumor expression of FcγR, non-selective membrane binding, and macropinocytosis *in cellulo.*

### Evaluation of MIL33B Targeting Tumor Cell 4Ig-B7-H3 Protein *In Vivo* and Application to Immuno-PET

Next, to determine if MIL33B could be utilized as an effective 4Ig-B7-H3 targeting antibody *in vivo* and demonstrate utility for radio-immuno PET applications, ^89^Zr-DFO conjugates of MIL33B and isotype control (^89^Zr-DFO-MIL33B and ^89^Zr-DFO-IgG2a, respectively) were generated and characterized. Live animal PET imaging allowed characterization of tumor-specific net retention, kinetics, and whole body biodistribution of MIL33B in different tumor models, and relative organ-specific clearance over time. Zirconium-89 was selected to exploit the long isotope half-life (3.3 days), which both aligned with the long half-life of circulating antibodies *in vivo* and provided ample time to conduct longitudinal imaging of mice injected with each radiolabeled antibody. Similar antibody conjugates have also been translated into humans for various PET applications 61.

**Figure 5:**
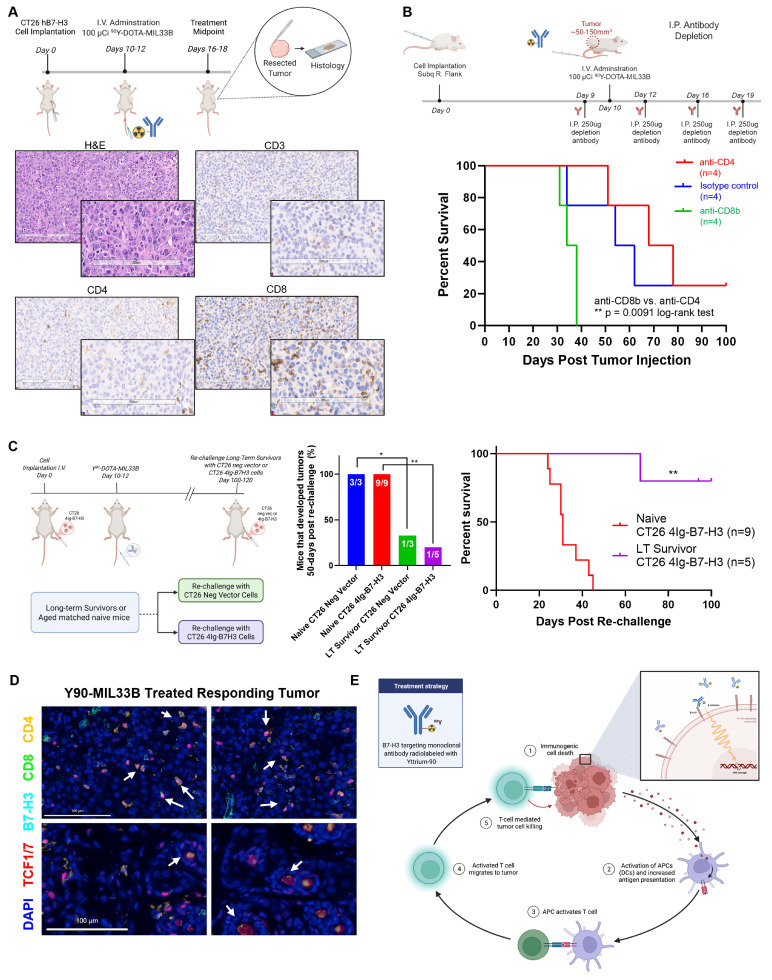
** Treatment with ^90^Y-DOTA-MIL33B elicits an immunological response. A.** Representative histology of a ^90^Y-DOTA-MIL33B treated tumor. Mice harboring established CT26 hB7-H3 tumors were treated with 100 μCi i.v. ^90^Y-DOTA-MIL33B. Six days post treatment mice were sacrificed and tumors were fixed in formalin and subsequently stored in 70% ethanol and allowed to decay. Histological staining demonstrated positive CD3, CD4 and CD8 staining in the tumor compartment indicating that both CD4 and CD8 T cells are present. Scale bars represent 200 μm. **B.** Immune cell depletion studies identify CD8b cells as essential for ^90^Y-DOTA-MIL33B response. Mice harboring CT26 4Ig-B7-H3 tumors were treated with 100 µCi of ^90^Y-DOTA-MIL33B i.v. One day before treatment and 2, 4, and 7 days after treatment mice received 250ug I.P. of either anti-CD4 or anti-CD8b depleting antibodies or the relevant isotype control antibody, rat isotype control. Survival curves were compared for isotype control (blue), anti-CD4 (red) and anti-CD8b (green) using the log-rank test. (** = p < 0.01).** C.** Re-challenge of ^90^Y-DOTA-MIL33B i.v. responsive mice. Mice harboring CT26 4Ig-B7-H3 tumors that were treated with 100 µCi of ^90^Y-DOTA-MIL33B i.v. and deemed long-term survivors were re-challenged with CT26 4Ig-B7-H3 cells or CT26 neg vector cells on the opposite flank 100-120 days after the initial tumor implantation. 4/5 of CT26 4Ig-B7-H3 and 2/3 of CT26 neg vector re-challenged mice rejected the tumors (at day 50 post-rechallenge). In contrast, all naïve mice grew tumors and reached endpoint. Survival curves were compared by the log-rank test (** = p < 0.006). **D.** Multiplexed immunofluorescence staining and microscopy of FFPE tissue extracted from a responding CT26 4Ig-B7-H3 model that was treated with 100 µCi of ^90^Y-DOTA-MIL33B i.v. was assessed for the presence of memory immune cells. CD4+ or CD8+ T-cells were identified with nuclear TCF1/7 staining (white arrows). Scale bar = 100 µm. **E.** Working model of the immunological response elicited by treatment of CT26-hB7-H3 tumors with ^90^Y-DOTA-MIL33B beta-radioligand therapy. Beta emissions induce cell damage, leading to immunogenic cell death, release of DAMPs and engagement of innate immunity (1). DAMPs activate APCs (2) leading to increased antigen presentation and activation of T cells (3). T cells then migrate to the tumor (4) and exert additional cytotoxic effects on tumor cells (5).

To synthesize ^89^Zr-DFO-MIL33B and ^89^Zr-DFO-IgG2a, each antibody was first conjugated to the chelator DFO **(Figure 5SA)**. LC/MS analysis of DFO-MIL33B and DFO-IgG2a identified a mass shift consistent with the addition of two DFO chelators on average per antibody **(Figure 5SD, 5SG)**. Subsequently, DFO-MIL33B and DFO-IgG2a were labeled with ^89^Zr-oxalate, purified on a PD-10 column, and characterized by radio-TLC and radio-SEC-HPLC. ^89^Zr-DFO-MIL33B labeled with 62.7% ± 13.7% (n = 4) chelation efficiency as measured by radio-TLC. Radio-SEC-HPLC of ^89^Zr-DFO-MIL33B immediately after purification demonstrated 82% purity and specific activity of 16.6 μCi/μg (6.14x10^-4^ GBq/μg) (n = 1) **(Figure 5SB-C)**. ^89^Zr-DFO-IgG2a labeled with 54.5% ± 14.2% (n = 4) chelation efficiency as measured by radio-TLC. Radio-SEC-HPLC of ^89^Zr-DFO-IgG2a immediately after purification demonstrated 87% purity and specific activity of 10 μCi/μg (3.7x10^-4^ GBq/μg) (n = 1) **(Figure 5SE-F)**.

After intravenous injection of either ^89^Zr-DFO-MIL33B or ^89^Zr-DFO-IgG2a, the biodistribution and 4Ig-B7-H3-specific tumor-targeting in nude mice harboring either HeLa 4Ig-B7-H3^+/+^ tumors or HeLa B7-H3^-/-^ tumors were evaluated by PET-CT at 24, 72, and 144 hours post injection. Furthermore, to evaluate the displaceability of ^89^Zr-DFO-MIL33B *in vivo,* some mice received 200 μg i.v. of non-radiolabeled MIL33B (cold block) 1 h before receiving ^89^Zr-DFO-MIL33B.

HeLa cells were again selected as a key test case because they endogenously express 4Ig-B7-H3, secrete s2IgB7-H3, and are infiltrated in the murine immune environment *in vivo* with MDSCs expressing FcγR 62, 63. ^89^Zr-DFO-MIL33B binding in the tumor compartment in mice harboring HeLa 4Ig-B7-H3^+/+^ tumors (4.27 ± 1.28 %ID/cc at 24 hours, 5.37 ± 1.40 %ID/cc at 72 hours, and 5.02 ± 1.30 %ID/cc at 144 hours post tracer injection; n = 3) was greater at all timepoints compared to mice harboring HeLa B7-H3^-/-^ tumors (1.77 ± 0.63 %ID/cc at 24 hours, 1.69 ± 0.68 %ID/cc at 72 hours, and 1.67 ± 0.61 %ID/cc at 144 hours post tracer injection; n = 3) **(Figure S6A-D)**. Note that both ^89^Zr-DFO-isotype control and pre-blocking experiments exhibited significantly different blood pool clearance times, necessitating individualized normalization to blood pool to test for 4Ig-B7-H3-specific tumor retention. To achieve this, organ and tumor activity (%ID/cc; SUV equivalent) was normalized to the blood pool activity (SUV heart) and expressed as organ to heart ratios (SUVR). Herein, blood normalized tumor retention in HeLa 4Ig-B7-H3^+/+^ tumors imaged with ^89^Zr-DFO-MIL33B (3.25 ± 0.65; n = 3) was significantly higher compared to HeLa B7-H3^-/-^ tumors (0.79 ± 0.13; n = 3; * p = 0.0201, un-paired two-tailed t-test), HeLa B7-H3^+/+^ tumors imaged with ^89^Zr-DFO-MIL33B after pre-blocking with cold MIL33B (1.02 ± 0.18; n = 3; * p = 0.0292, un-paired two-tailed t-test), HeLa 4Ig-B7-H3^+/+^ tumors imaged with ^89^Zr-DFO-IgG2a (0.77 ± 0.15; n = 3; * p = 0.0201, un-paired two-tailed t-test), or HeLa B7-H3^-/-^ tumors imaged with ^89^Zr-DFO-IgG2a (0.67 ± 0.13; n = 3; * p = 0.0173, un-paired two-tailed t-test) **(Figure 3A, 3C)**. In 4Ig-B7-H3-expressing tumors, tumor activity increased over 24 - 144 hours, whereas the blood pool and other organs showed tracer washout over time **(Figure S7A, 7SC)**. Tumor sizes for HeLa 4Ig-B7-H3^+/+^ tumors imaged with ^89^Zr-DFO-MIL33B ranged from 32 mm^3^ - 275.7 mm^3^ (mean: 159.7 mm^3^ SD +/- 122.3 mm^3^), compared to HeLa 4Ig-B7-H3^-/-^ tumors, which ranged from 92.26 mm^3^ - 453.63 mm^3^ (mean: 273.8 mm^3^ SD +/- 180.7 mm^3^) **(Figure S7)**. Tumor retention of ^89^Zr-DFO-MIL33B did not correlate with tumor size **(Figure S7D)**. These results demonstrated that ^89^Zr-DFO-MIL33B was specific for human 4Ig-B7-H3 in the tumor compartment *in vivo* versus normal tissues or other tumor antigens and demonstrated the requirement for 4Ig-B7-H3 antigen-specific CDRs for tumor retention.

Robustness was further validated by testing 4Ig-B7-H3-expressing 4T1, CT26, and B16F10 syngeneic tumor models. These tumors represent two highly immune infiltrated models and one “immune desert” model (B16F10), respectively, and furthermore enabled assessment of targeting across TH1 (C57BL/6) and TH2 (Balb/c) murine backgrounds 64. Balb/c mice were implanted subcutaneously with 4T1 4Ig-B7-H3 cells on the right flank and 4T1 negative vector cells on the left flank, and subsequently injected i.v. with either ^89^Zr-DFO-MIL33B or ^89^Zr-DFO-IgG2a prior to imaging by PET-CT at 24, 72, and 144 hours post tracer injection. Similar to the HeLa tumor model, statistically significant greater retention of ^89^Zr-DFO-MIL33B was observed in 4T1 4Ig-B7-H3 tumors compared to 4T1 negative vector tumors, or retention of ^89^Zr-DFO-IgG2a in 4T1 4Ig-B7-H3 tumors, or ^89^Zr-DFO-IgG2a in 4T1 in neg vector tumors **(Figure S6E-H).** When tumor-specific retention at 72 hours post tracer injection was normalized to blood pool (heart-associated) counts and compared across tumor types and antibodies, significantly higher normalized retention of ^89^Zr-DFO-MIL33B in 4T1 4Ig-B7-H3 tumors (2.16 ± 0.19) was observed compared to the retention of ^89^Zr-DFO-IgG2a in 4T1 4Ig-B7-H3 tumors (0.64 ± 0.16, ** p 0.0036, un-paired two-tailed t-test) or in 4T1 neg vector tumors (1.07 ± 0.18, * p = 0.0135, un-paired t-test) **(Figure 3E)**. Although a decrease in the average normalized tumor retention of ^89^Zr-DFO-MIL33B in 4T1 negative vector tumors was seen compared to 4T1 4Ig-B7-H3 tumors, the effect size was small. Nonetheless, these results demonstrated overall that expression of human 4Ig-B7-H3 in 4T1 tumors significantly increased tumor-specific retention of ^89^Zr-DFO-MIL33B *in vivo* relative to isotype control, and CDR-specific binding to the tumor compartment in a highly immune infiltrated environment, replete with FcγR expression and internalizing MDSCs 63, 65.

Next, the biodistribution and tumor specific-retention of ^89^Zr-DFO-MIL33B and ^89^Zr-DFO-IgG2a in mice harboring B16F10 (immune desert) tumors either transduced with human 4Ig-B7-H3 or transfected with the relevant negative vector control was evaluated. Statistically significant greater tumor retention of ^89^Zr-DFO-MIL33B in B16F10 4Ig-B7-H3 tumors was again observed compared to B16F10 negative vector tumors, or tumor retention of ^89^Zr-DFO-IgG2a in B16F10 4Ig-B7-H3 tumors or B16F10 negative vector tumors **(Figure S6I-K)**. When blood pool normalized, there was significantly higher normalized tumor retention of ^89^Zr-DFO-MIL33B in B16F10 4Ig-B7-H3 tumors (3.16 ± 0.30; n = 5) compared to B16F10 negative vector tumors (1.20 ± 0.69; n = 3; ** p = 0.0032, un-paired two-tailed t-test), retention of ^89^Zr-DFO-IgG2a in B16F10 4Ig-B7-H3 tumors (0.81 ± 0.41; n = 4; *** p = 0.0003, un-paired two-tailed t-test) or in B16F10 neg vector tumors (0.89 ± 0.52; n = 3; ** p = 0.0015, un-paired two-tailed t-test). These results demonstrated that expression of human 4Ig-B7-H3 in the B16F10 murine melanoma model significantly increased tumor-specific retention of MIL33B *in vivo* **(Figure 3F)**.

Finally, the biodistribution and tumor-specific retention of ^89^Zr-DFO-MIL33B and ^89^Zr-DFO-IgG2a in the CT26 murine colon cancer model was investigated. Mice were implanted subcutaneously on the right flank with either CT26 4Ig-B7-H3 cells or CT26 negative vector cells and imaged by PET-CT at 24 and 72 hours post tracer injection (i.v.). As before, following blood pool normalization, significantly higher tumor-specific retention of ^89^Zr-DFO-MIL33B was found in CT26 4Ig-B7-H3 tumors (3.62 ± 1.62; n = 5) compared to CT26 negative vector tumors (1.89 ± 0.85; n = 5; *** p < 0.0001, un-paired two-tailed t-test), or tumor retention of ^89^Zr-DFO-IgG2a in CT26 4Ig-B7-H3 tumors (1.59 ± 0.71; n = 5; *** p < 0.0001, un-paired two-tailed t-test) or in CT26 negative vector tumors (1.32 ± 0.59, tumors; n = 5; *** p < 0.0001, un-paired two-tailed t-test) **(Figure 3B, 3D, Figure S6-7S)**. Again, in this model, these results demonstrated that expression of human 4Ig-B7-H3 significantly increased tumor-specific retention of MIL33B *in vivo*.

### MIL33B as a 4Ig-B7-H3-Targeted Radio-Ligand Therapeutic

After validating *in vivo* the 4Ig-B7-H3-specific tumor-targeting properties of MIL33B by PET-CT*,* MIL33B was engineered into an antibody radio-conjugate for the treatment of solid tumors. Yttrium-90-DOTA conjugates of MIL33B were generated to exploit a beta-emitting radionuclide used clinically in other radio-immunotherapies 66-68 **(Figure 1B)**.

MIL33B was first conjugated to DOTA, followed by chelation of ^90^Y **(Figure 4A, Figure S8)**. LC/MS of DOTA-MIL33B was consistent with a mixed pool of one to zero DOTA chelators per antibody **(Figure S8C-D)**. MIL33B-DOTA labeled with ^90^Y at greater than 61.9% ± 17.2% (n = 4) chelation efficiency as determined by radio-TLC. Radio-SEC-HPLC of purified ^90^Y-DOTA-MIL33B demonstrated > 99% purity (n = 5) and a specific activity of 3.15 ± 0.50 μCi/μg (1.17x10^-4^ GBq/μg) (n = 4) **(Figure 4B and Figure S8A-B)**.

### Efficacy of Beta-RLT on 4Ig-B7-H3-Expressing HeLa Tumor Xenografts *In Vivo*

As with fluorescence and ^89^Zr-PET imaging, RLT efficacy was first tested with human HeLa xenografts in immunocompromised mice to generate preliminary signals of efficacy in a model that secretes soluble 2Ig-B7-H3 **(Figure S9)**. Mice harboring HeLa 4Ig-B7-H3^+/+^ tumors (n = 10) or mice harboring HeLa B7-H3^-/-^ KO tumors (n = 7) received a single dose (100 µCi) of ^90^Y-DOTA-MIL33B 30 days after tumor implantation and followed for 120 days **(Figure 4C, Figure S10)**. Compared with untreated animals harboring HeLa WT and KO xenografts **(Figure 4D-F)**, a single dose of ^90^Y-DOTA-MIL33B modestly extended median survival for WT B7-H3 expressing tumor xenografts, where the tumors expressed 4Ig-B7-H3 (*** P < 0.001, log-rank test; 0.2706, hazard ratio ^90^Y-DOTA-MIL33B/no Tx, 95% CI ratio: 0.1140 to 0.6423) **(Figure 4D)**. There was no significant survival benefit for mice harboring B7-H3 KO tumors treated with ^90^Y-DOTA-MIL33B, indicating a genotype-specific response **(Figure 4E)**. When all survival curves were superimposed **(Figure 4F)**, the magnitude of response by a single dose RLT can be appreciated wherein ^90^Y-DOTA-MIL33B-RLT extended median survival in this immunocompromised model. For a more detailed examination, modified interrupted time series analysis was conducted to compare individual growth rates 15 days before treatment and 15 days after treatment on a per tumor basis. There was a significant decrease in tumor growth rates in treated HeLa 4Ig-B7-H3^+/+^ tumors (2-way ANOVA, ** p = 0.0019) as compared to HeLa B7-H3^-/-^ KO tumors (2-way ANOVA, p = 0.1027) whereby B7-H3 expression on the tumor was knocked out, demonstrating an initial B7-H3-targeted treatment response to ^90^Y-DOTA-MIL33B **(Figure S10C-D).** Note that knockout of B7-H3 in HeLa cells substantially shifts their growth rates compared to wild type cells, the mechanism by which has been dissected by Sutton et. al. 69, 70. However, compared to short term effects (15 days), substantial long-term differences in overall survival at the end point of paired RLT treatment were not observed **(Figure S10A-B)**. In this immuno-compromised model, an eventual rebound in tumor growth following single dose treatment was observed, consistent with a lack of engagement of the adaptive immune system.

### Efficacy of Beta-RLT in a Syngeneic Tumor Model *In Vivo*

Next, syngeneic immune-competent models of RLT were tested. To fully evaluate the therapeutic efficacy of intravenous ^90^Y-DOTA-MIL33B, mouse cohorts harboring established CT26 4Ig-B7-H3 or negative vector tumors were tumor-size selected (50-150 mm^3^) 10 to 12 days after cell implantation **(Figure 4C)**. Untreated mice were followed regardless of initial tumor size. Based on the results of a pilot dose-response trial, a single dose (100 µCi) of ^90^Y-DOTA-MIL33B i.v. was characterized in detail for 4Ig-B7-H3-based therapeutic responses. Overall, from a combination of five independent experimental cohorts, 15 mice harboring CT26 4Ig-B7-H3 tumors received ^90^Y-DOTA-MIL33B i.v., 9 mice harboring CT26 negative vector tumors received ^90^Y-DOTA-MIL33B i.v., 23 mice harboring CT26 4Ig-B7-H3 tumors received an equivalent volume of saline i.v., and 20 mice harboring CT26 4Ig-B7-H3 tumors received no treatment. In the CT26 4Ig-B7-H3 group that received a *single* dose of 100 µCi ^90^Y-DOTA-MIL33B i.v., an initial tumor regression of 11 out of 15 tumors was observed; however, 3 tumors emerged at later time points. In the CT26 negative vector group that received 100 µCi ^90^Y-DOTA-MIL33B i.v., an initial tumor regression of 2 out of 9 tumors was observed and 1 of these tumors later emerged. In the CT26 4Ig-B7-H3 group that received intravenous saline, tumor regression of only 3 out of 23 tumors was found, and in the CT26 4Ig-B7-H3 groups that did not receive any treatment, spontaneous tumor regression of 1 out of 20 tumors was observed **(Figure S11A-D)**. When mice were followed for 100 days, 53% long-term survivors were observed in mice harboring CT26 4Ig-B7-H3 tumors treated with ^90^Y-DOTA-MIL33B i.v., compared to only 11% long-term survivors in mice harboring CT26 negative vector tumors receiving the same dose (* p = 0.0376, log-rank test), 13% long-term survivors in mice harboring CT26 4Ig-B7-H3 tumors treated with saline (** p = 0.0034 , log-rank test), and 5% long-term survivors in mice harboring CT26 4Ig-B7-H3 tumors that received no treatment (*** p = 0.0003, log-rank test) **(Figure 4G)**. When tumors in a subset of animals were harvested at the typical *mid-point* of treatment (day 6-8 post initial ^90^Y-DOTA-MIL33B injection), representative tumors from mice harboring CT26 4Ig-B7-H3 tumors treated with ^90^Y-DOTA-MIL33B were significantly smaller compared to untreated mice harboring the same tumors **(Figure 4H)**.

The low yield positron emission of^ 90^Y (71) is below the threshold for detection by conventional animal PET imaging at the dilute, yet therapeutic, concentrations of ^90^Y tested herein 72. As an alternative to PET imaging, Cherenkov radiation emitted by the beta decay was observed by optical imaging to non-invasively qualify net retention of ^90^Y-DOTA-MIL33B in the tumor compartment longitudinally, which confirmed preferential localization of ^90^Y-DOTA-MIL33B to 4Ig-B7-H3 tumors versus control tumors *in vivo*** (Figure 4I)**, similar to that observed by PET-CT with ^89^Zr-labeled MIL33B. Furthermore, white light mode imaging allowed observation of individual tumors over time, confirming complete regression of representative CT26 4Ig-B7-H3 tumors and continued growth of CT26 negative vector tumors **(Figure 4I).** In addition to complete tumor regression, no obvious radiotoxicity was observed in mice that responded to treatment with ^90^Y-DOTA-MIL33B (weight, fur, diet consumption, and activity were grossly normal as monitored both by the investigators and impartial veterinary staff). These results demonstrated that a single i.v. injection of ^90^Y-DOTA-MIL33B led to complete regression of over half of established CT26 murine colorectal tumors in a 4Ig-B7-H3-dependent manner, demonstrating promise for a high therapeutic index.

To assess the potential impact of 4Ig-B7-H3 neo-antigens as the dominant immune driver, RNA sequencing was performed on the CT26 4Ig-B7-H3 cell line and compared to CT26 empty vector control cells. Gene expression data revealed significantly concordant gene expression profiles (Lin's CCC = 0.9817), with tightly overlapping gene density profiles **(Figure 4J)**. Similarly, tumor growth rates and scRNA-Seq data were obtained from untreated B16F10 tumors expressing the exact same empty vector control or human 4Ig-B7-H3 constructs as the CT26 tumors. Overall tumor growth rates were highly correlated, and UMAP plots of 4Ig-B7-H3-expressing B16F10 versus vector control tumors demonstrated strong concordance for the immune cell infiltrates and subtype composition numbers between the two tumor types **(Figure S12)**. These data supported the lack of human 4Ig-B7-H3 neo-antigens alone as the dominant immune driver accounting for the response to MIL33B-RLT therapy.

### Non-Radiolabeled (Cold) MIL33B Therapy

As an additional control, MIL33B efficacy as a single agent antibody was explored at a mass in vast excess relative to RLT in a manner consistent with established immunotherapy regimens for other B7-family targeted anti-cancer therapies. This experiment served as a control to estimate the potential therapeutic contribution of binding of cold MIL33B to human 4Ig-B7-H3, acting either through blockade of B7-H3 signaling pathways, complement, ADCP, or ADCC. Mice implanted with CT26 4Ig-B7-H3 tumors received either 200 μg of MIL33B (non-radiolabeled) i.p., 200 μg of mouse IgG2a isotype control i.p., or PBS i.p., every three days post tumor cell implantation until endpoint **(Figure S13A)**, a typical regimen for immune checkpoint therapy in pre-clinical models 73. Note that immune checkpoint regimens involve substantially higher therapeutic mass of MIL33B (> 32-fold) than the mass used for a single administration of ^90^Y-DOTA-MIL33B (≤ 50 μg). Compared to ^90^Y-DOTA-MIL33B therapy, treatment with cold MIL33B was far inferior, showing no observable effect in this tumor model **(Figure S13A-D).**

### ^90^Y-DOTA-MIL33B Engages Adaptive Immunity

After establishing that 100 µCi of ^90^Y-DOTA-MIL33B i.v. led to differential regression of established CT26 tumors that expressed human 4Ig-B7-H3 compared to CT26 tumors with low B7-H3 expression, the mechanism of action of this radio-therapeutic response was assessed. First, the composition of the TIME in mice treated with ^90^Y-DOTA-MIL33B was characterized by histology. Tumors from mice harboring CT26 4Ig-B7-H3 tumors that had received 100 µCi of ^90^Y-DOTA-MIL33B i.v. were excised at the midpoint of treatment, fixed and saved for histology after radioactive decay **(Figure 5A)**. Representative IHC tumor staining for CD3^+^, CD4^+^, and CD8^+^ antigens demonstrated positive staining for each of these markers inside the tumor compartment, indicating that CD8^+^ and CD4^+^ T cells had widely infiltrated into the TIME in mice treated with ^90^Y-DOTA-MIL33B i.v. **(Figure 5A).** In addition, the CD3^+^, CD8^+^, and CD4^+^ cell populations tended to co-correlate across groups **(Figure S14)**.

Having validated antibody binding to tumor-associated 4Ig-B7-H3, mechanistic intervention studies were conducted. Because CD8^+^ and CD4^+^ T cells were present in the TIME in mice that had previously received 100 µCi of ^90^Y-DOTA-MIL33B i.v., the dependence of treatment response on components of the immune system downstream of the initial radio-ablation was further evaluated. Herein, mice harboring CT26 4Ig-B7-H3 tumors received intravenous treatment with 100 µCi of ^90^Y-DOTA-MIL33B as described previously. However, one day before treatment, mice received 250 µg i.p. of either anti-CD4- or anti-CD8b-depleting antibodies or the relevant isotype control antibody (anti-rat isotype antibody). Mice subsequently received i.p. injections of depleting antibodies or relevant control antibodies on day 2, day 6, and day 9 post treatment **(Figure 5B)**. For mice that received anti-CD4-depleting antibodies, 3 out of 4 tumors initially regressed, but later, 2 of those 3 tumors reemerged. In mice that received anti-CD8b-depleting antibodies, all tumors progressed, and in mice that received anti-rat isotype control antibodies 1 out of 4 tumors regressed **(Figure S15A-C)**. When mice were followed for 100 days post tumor implantation, no mice in the group that received anti-CD8b-depleting antibody plus 100 µCi of ^90^Y-DOTA-MIL33B were deemed long-term survivors and these mice reached endpoint faster than mice that received anti-CD4-depleting antibodies plus 100 µCi of ^90^Y-DOTA-MIL33B (25% long-term survivors; ** p = 0.0091, log-rank test). In addition, injections of rat isotype control did not affect the therapeutic efficacy of 100 µCi of ^90^Y-DOTA-MIL33B (25% long-term survivors) **(Figure 5B).** Although relatively smaller sample sizes were used, statistical significance was achieved. Thus, these results indicated that CD8b+-expressing cells, but not CD4+-expressing cells, were necessary to confer long-term therapeutic effects of ^90^Y-DOTA-MIL33B *in vivo* and pointed to CD8+ T cells or CD8+ NKT cells as essential contributors to long-term survival.

### Documenting Immunological Memory

While both the HeLa and CT26 models required human 4Ig-B7-H3 in the tumor compartment for beta-RLT to induce an early decrease in tumor volumes, long-term cures were primarily evident in the immune-competent CT26 system. Indeed, there was a correlation between response rate and adaptive immune engagement to produce long-term survivors; nude (nu/nu) mice had 1 long-term survivor whereas syngeneic models that had the ability to engage both innate and adaptive immunity had 8/15 cures. These data and the observed RLT-induced tumor immune infiltrates suggested a potent mechanistic role for cellular adaptive immunity. Thus, to test the potential for cured mice to develop immunological memory as a result of MIL33B-RLT treatment, mice harboring CT26 4Ig-B7-H3 tumors previously treated with 100 µCi of ^90^Y-DOTA-MIL33B and deemed long-term survivors (90 to 110 days post treatment), received a second re-challenge of fresh CT26 4Ig-B7-H3 cells or CT26 neg vector cells implanted on the left (opposite) flank. Re-challenged mice were compared to naïve mice (age matched; never before inoculated with tumor cells nor treated) also implanted subcutaneously with matched fresh CT26 4Ig-B7-H3 or CT26 negative vector cells on the left flank **(Figure 5C)**. Both cohorts were followed for 100 days post tumor cell implantation (200+ days from initial tumor implantation). At study end point, in a combination of two independent experiments, all naïve mice had grown tumors and reached terminal endpoint. Only 1 out of the 5 CT26 4Ig-B7-H3 tumor cell re-challenged mice grew a tumor (** p = 0.0035, log-rank test); the remaining 4 re-challenged mice were able to fend off tumor re-challenge and survive **(Figure 5C)**. Similarly, in the previously treated cohort, 2/3 mice (66%) implanted with CT26 neg vector cells were also able to fend off tumor regrowth. These results indicated that mice with CT26 4Ig-B7-H3 tumors that had previously responded to radio-ligand therapy developed immunological memory, a signature indicating that adaptive immunity was indeed engaged for long-term survival. Additionally, the ability for the re-challenged mice to fend off both CT26-4Ig-B7-H3 and CT26 negative vector tumors suggested that human 4Ig-B7-H3 neo-antigens alone were not the dominant immune driver.

Additionally, multiplexed immunofluorescence staining revealed that many CD4- and CD8-positive immune cells in the TIME of CT26 4Ig-B7-H3 tumors responding to ^90^Y-DOTA-MIL33B were also positive for nuclear staining of TCF1/7. The presence of nuclear TCF1/7 staining within CD4⁺ and CD8⁺ T-cell populations indicate a stem-like memory phenotype 74, which is critical for sustaining long-term antitumor immunity. Additionally, germinal center structures exhibited dense nuclear TCF1/7 positivity, consistent with active reservoirs of progenitor-like T cells capable of self-renewal and differentiation upon antigen re-exposure **(Figure 5D)**. Overall, these findings supported the engagement of durable adaptive immune mechanisms following RLT and the proposed model wherein the effects of ^90^Y-DOTA-MIL33B were due to 4Ig-B7-H3-mediated retention of a therapeutic radionuclide, yttrium-90, within the local tumor environment that initiated immunogenic cell death, leading to engagement of innate immunity, an immune priming cascade, and ultimately activation of adaptive immunity **(Figure 5E).**

## Discussion

While B7-H3 expression is generally thought to be “pro-tumor” and immunosuppressive in humans, the detailed signaling functions of the 4Ig and 2Ig isoforms of B7-H3 remain under investigation with both co-stimulatory and co-suppressive immune effects being reported both in cancer and in other inflammatory disorders 40, 75-89. Indeed, the complexities may be species-specific as rodents have evolutionarily lost the 4Ig isoform. Thus far, non-isoform selective anti-B7-H3 antibody binding or blocking strategies have met with limited clinical success in oncology, particularly relative to anti-B7 therapies targeting the PD-1/PD-L1 axis, although combination anti-B7-H3 therapy with anti-PD-L1 in the context of *PTEN*/*TP53-*deficiency shows promise in pre-clinical prostate cancer and AML models 90, 91. Interestingly, soluble ectodomain antigens (in this case, shed ectodomain 2Ig-B7-H3) present challenges to antibody therapy because antibodies bound to antigens are often cleared faster from circulation, altering pharmacokinetics 53, 92, 93. Furthermore, antibody antigen complexes can lead to off-target toxicities both in the local micro-environment, and in FcγR2-expressing cells 94, 95. These off-tumor effects can be further compounded in the case of B7-H3 because the concentration of circulating s2Ig-B7-H3 can increase with tumor progression or advanced inflammation, dynamically impacting dosing regimens or potential toxicities 49, 96-100.

Herein, a new high affinity, isoform-selective anti-4Ig-B7-H3 antibody was selected through early use of live cell binding and imaging *in vivo* using strategic models known to present other confounders of binding, including possible tumor-specific glycosylation 101, 102 and dimerization 69, 70. Through early use of live cell selection, B_max_ (the maximum specific binding) in addition to K_d_ could be subtly maximized through selection for antibody binding epitopes that are accessible in the context of folded proteins, quaternary structure, and cell surface supramolecular architectures. Isoform-selective anti-4Ig-B7-H3 binding was characterized and validated through biochemical assays *in vitro*, live cell binding and microscopy assays with knockout, knock- in, and blocking studies *in cellulo*, and similarly executed tumor binding and PET-CT imaging studies *in vivo*, which were robust to multiple cell lines, immune-competent and immune-defective murine models, and using both human cells as well as 4Ig-B7-H3 transfected mouse cells. Non-specific membrane adhesion, macro-pinocytosis, B7 paralogues, tumor surface antigens, tumor-expressed FcγR binding, blood accessible murine surface antigens *in vivo*, FcγR binding *in vivo*, and enhanced permeability-retention (EPR) *in vivo* were tested and demonstrated non-contributory. Finally, through this development process, the platform utility of the antibody for systemic delivery of cargo to 4Ig-B7-H3 compartments *in vivo*, such as the TIME, was demonstrated with PET-CT imaging, Cherenkov imaging, and critically, beta-RLT in a syngeneic CT26 tumor model. Of note, CT26 tumors are characterized as an aggressive MSI-low colorectal carcinoma model radioresistant to conventional external beam radiotherapy 73-75, thus, setting a high bar for pre-clinical advancement of beta-RLT 103-105.

Further analysis of the TIME of CT26 4Ig-B7-H3 tumors at the midpoint of beta-RLT with ^90^Y-DOTA-MIL33B revealed that CD4^+^ and CD8^+^ T cells had infiltrated into the TIME. In addition, mice harboring established CT26 4Ig-B7-H3 whose tumors had previously regressed in response to ^90^Y-DOTA-MIL33B developed immunological memory. These phenotypes generally depend upon activation of adaptive immunity. To test this proposed mechanism of immune memory and cure, *in vivo* depletion assays demonstrated that the final therapeutic efficacy of ^90^Y-DOTA-MIL33B depended upon CD8b^+^-expressing cells. These results led to a working model wherein the therapeutic effects of ^90^Y-DOTA-MIL33B rely on early radiation-induced initiation of immunogenic cell death, activation of innate immunity and antigen presentation, leading to secondary adaptive immune responses and activation of cytotoxic T cells. This is further supported by the early differential response to treatment observed in an immunocompromised tumor model of mice bearing established human HeLa B7-H3^+/+^ or HeLa B7-H3^-/-^ tumors, wherein treatment with a single dose of ^90^Y-DOTA-MIL33B resulted in a significant initial regression of HeLa B7-H3^+/+^-treated tumors superior to HeLa B7-H3^-/-^ tumors. In addition, median survival of ^90^Y-DOTA-MIL33B-treated HeLa B7-H3^+/+^ tumors showed a significant right-shift consistent with a model wherein RLT with ^90^Y-DOTA-MIL33B provided an initial immune priming event that could not further engage T-cell mediated immunity in nude mice.

These results point to the general potential of beta-emission-based 4Ig-B7-H3-targeted RLT or radioimmunotherapy as immune priming agents. Previous work has implicated two pathways in radiation-induced activation of the adaption immune system, the release of DAMPs and activation of the cGAS-STING pathway 106, 107. Both pathways can stimulate antigen presenting cells to present neo-antigens to T cells. Further work with relevant knockout mouse models may pinpoint signaling pathways activated by ^90^Y-DOTA-MIL33B and additional pharmacodynamic biomarkers downstream of target engagement. In this regard, it is interesting to speculate that the cross-fire properties of beta-emitting therapeutic radionuclides such as ^90^Y (and ^177^Lu, ^67^Cu) may enhance the repertoire of neoantigens released within the TIME, since both 4Ig-B7-H3-expressing and nearby non-expressing cells will be impacted by local beta emissions, perhaps overcoming intercellular genetic heterogeneity and resistance mechanisms. In addition, although T cells were present in the TIME after treatment with ^90^Y-DOTA-MIL33B, it was unknown whether ^90^Y-DOTA-MIL33B actively recruited peripheral T cells to the TIME or if ^90^Y-DOTA-MIL33B changed the activation setpoint of T cells already residing in the TIME. Discerning this difference may help to further elucidate the mechanism of action of ^90^Y-DOTA-MIL33B as well as understand the milieu of the TIME most responsive to RLT. Furthermore, it would be predicted that early disease or micro-metastases will also be impacted by beta-RLT, enabling systemic treatment of metastatic disease, in contrast to EBRT, generally limited to local control of a primary tumor or oligometastatic disease **(Figure 1A-B)**.

Development of an isoform-selective anti-4Ig-B7-H3 antibody poses a challenge for use of murine models for characterization of a human-restricted protein target. Mice only express 2Ig-B7-H3, and it could be argued that the effects observed in our study were dominated by human 4Ig-B7-H3 representing a neo-antigen in the context of murine tumor models. However, the ability for the re-challenged mice to fend off both CT26-4Ig-B7-H3 and CT26 neg vector tumors suggested that human 4Ig-B7-H3 neo-antigens were not the dominant immune driver. In addition, tumors treated with non-radioactive MIL33B in a regimen similar to that used with standard immune checkpoint therapies (anti-PD-L1/PD-1; anti-CTLA4), even at much higher “mass” concentrations, showed no significant effect, similar to those treated with IgG2a isotype control antibody or PBS. These data not only provide evidence that MIL33B antibody *per se* was not working in an immune blockade/therapeutic fashion on its own, but highlight that the delivery of beta radioactivity was promoting the observed anti-tumor effect. Finally, using untreated 4Ig-B7-H3-expressing B16F10 tumors versus vector control tumors expressing the same constructs used for all models, scRNA-Seq analysis and UMAP plots demonstrated strong concordance for the identified immune cell infiltrates and numbers of each cell type, further supporting the lack of human 4Ig-B7-H3 neo-antigens alone as the dominant immune driver.

Clinically, radioembolization with ^90^Y in the treatment of hepatic malignancies has previously been shown to increase the CD8^+^ T-cell and NKT-cell infiltration into the TIME 108. Pre-clinically, others have demonstrated that ^90^Y-based radionuclide therapy can activate the adaptive immune system in a Non-Hodgkin's Lymphoma model and synergize with immunotherapy to increase CD8^+^ T-cells in the TIME of pre-clinical solid tumor models 109, 110. ^90^Y-radio-embolization can elicit an adaptive immune response in humans 108, and human anti-mouse antibody reactions can predict survival after radio-immunotherapy in humans 111-113. Immune activation downstream of ^90^Y-DOTA-MIL33B treatment point to the clinical potential of 4Ig-B7-H3-targeted RLT as both a stand-alone agent and potential immune priming agent for use in combination with other immune checkpoint or immunotherapies for the treatment of many 4Ig-B7-H3-expressing solid and liquid tumors.

B7-H3 is under active exploration as an immune checkpoint, ADC, and CAR T-cell target, and has been previously explored as a radio-immunotherapy target 25, 34-36, 114. Pre-clinically, ^212^Pb-376.96 has been explored for the treatment of human ovarian cancer xenografts and ^131^I-4H7 has been investigated in the treatment of human renal cell carcinoma xenografts 115-117. These pre-clinical studies demonstrated either extended overall survival or tumor growth rate inhibition 115-117. However, these pre-clinical studies mainly utilized immunodeficient models, and not immunocompetent models, thus curtailing the full range of mechanism of action or resistance to a radio-immunotherapeutics when both the innate and adaptive immune systems are present. 8H9, a murine anti-B7-H3 antibody, is the only anti-B7-H3 RLT that has been investigated clinically 54, 118, focused on non-humanized ^131^I-8H9 for the treatment of metastatic neuroblastoma or medulloblastoma by intrathecal injection. ^131^I-8H9 and ^123^I-8H9 are also under investigation for the treatment of CNS-relapsed rhabdomyosarcoma and DIPG, respectively 119-123. These trials have yielded promising results in closed CNS compartments 119-123, but attempts at systemic therapy of solid tumors have not been reported, perhaps related to the overall lower affinity of the primary antibody as well as the lack of differential isoform selectivity of 8H9, which has higher affinity for the human 2Ig isoform compared to the 4Ig isoform. The selective 2Ig-B7-H3 affinity may be sufficient when administered in a closed compartment such as the CNS; however, systemic therapy will be substantially hindered by the soluble 2Ig decoy. Similarly, differential uptake was observed by Fernandes et. al. 114 when comparing two anti-B7-H3 antibodies with differences in isoform affinity, highlighting the importance of understanding both tumor biology and circulating soluble decoys to optimize tumor-specific targeting. This is especially important in the context of systemic RLT, wherein high specific activity radiolabeling methods require use of low masses of antibody (compared to masses of antibody used with immune checkpoint or ADCs). Low mass regime therapeutics, such as RLT, utilizing antibodies with poor selectivity will bind blood decoys, form micro- and macro-immune complexes, remain in circulation or deposit in tissues 124, and thus, deplete the primary antibody, adversely impact pharmacokinetics, and abrogate therapeutic effectiveness. Indeed, soluble decoys may confound many targets relevant to the RLT field, including most B7 family members, as well as PSMA, EGFR, and IGF1R 125-129. The promise of pre-clearing and pre-targeting strategies used in RLT to manage decoy targets can be a two-edged sword because of immune complex formation 130. Traversing soluble decoys through engineered antibodies as demonstrated herein provides an alternative and/or complementary strategy.

## Conclusions

In summary, ^90^Y-DOTA-MIL33B is an effective radio-ligand therapeutic for the treatment of 4Ig-B7-H3-expressing solid tumors and may have broad potential both as a stand-alone RLT agent and in combination with other immune modulators. Because MIL33B is a high affinity 4Ig-B7-H3-specific targeting agent engineered to traverse soluble (shed ectodomain) s2Ig-B7-H3 *in vivo*, MIL33B provides a modular platform of CDRs and antibody fragments for the delivery of additional therapeutic cargoes, including other beta-emitting and alpha-emitting isotopes, antibody-drug conjugates, peptides, bi- or tri-specific antibodies, and cell-based therapies in oncology. Additional cell-based therapeutics and applications may apply to cardiovascular disease, inflammation, and rheumatological disorders engaging 4Ig-B7-H3, including immune-depletion of memory cells in autoimmunity 131, 132.

## Supplementary Material

Supplementary data, methods, figures and tables.

## Figures and Tables

**Table 1:** ** Comparative Affinity and Selectivity of MIL33B to B7-H3 Isoforms.** Biolayer interferometry analysis of MIL33B binding to the extracellular domains of human 4Ig-B7-H3, human 2Ig-B7-H3, mouse 2Ig-B7-H3, and porcine 4Ig-B7-H3. MIL33B demonstrated higher affinity and higher selectivity for the human 4Ig isoform over the 2Ig isoform when compared to other anti-B7-H3 antibodies that have been tested clinically (NR = not reported or calculable). Affinities from other antibodies collected from published values 33, 54, 55, 140, 141. ADC = antibody-drug conjugate, ADCC =antibody dependent cellular cytotoxicity, UT MDACC = University of Texas MD Anderson Cancer Center, MSKCC = Memorial Sloan Kettering Cancer Center, PUCHI = Peking University Cancer Hospital and Institute.

*Antibody*	*Protein*	*K_D_ (M)*	*K_D_ Error*	*Full R^2^*	*Affinity ratio: 4Ig to 2Ig*
***MIL33B*** *Source: UT MDACC* *Mechanism of Action: RLT & others to be determined*	Human 4Ig-B7-H3	7.23E-11	5.96E-12	0.9962	
	Human 2Ig-B7-H3	5.80E-10	5.17E-11	0.9535	8
	Mouse 2Ig-B7-H3	4.11E-08	2.98E-09	0.93	18
	Porcine 4Ig-B7-H3	1.02E-10	1.45E-11	0.99	
***Murine 8H9*** *Source: MSKCC, Y-mAbs Therapeutics* *Mechanism of Action: RLT & inhibition*	Human 4Ig-B7-H3	8.90E-09			
	Human 2Ig-B7-H3	8.10E-10			0.09
***MGA271*** *Source: Macrogenics* *Mechanism of Action: RLT, ADCC, CAR-T using scFV*	Human 4Ig-B7-H3	9.50E-09			NR
***DS-7300 (Hu-M30)*** *Source: Daiichi Sankyo* *Mechanism of Action: ADC*	Human 4Ig-B7H3	1.60E-09			
	Human 2Ig-B7-H3	1.60E-08			10.0
***ABBV-155 (huAb13v1)*** *Source: AbbVie* *Mechanism of Action: ADC*	Human (isoform not reported)	6.20E-09			NR
***MGC018*** *Source: Macrogenics* *Mechanism of Action: ADC*	Human 4Ig-B7H3	2.20E-08			NR
***Affibody-BCH*** *Source: PUCHI* *Mechanism of Action: RLT*	Human 4Ig-B7-H3	4.5E-9			NR
***Hu4G4*** *Source: Soochow University* *Mechanism of Action: RLT*	Human 4Ig-B7-H3	9.9E-10			NR

**Table 2:** ** MIL33B Affinity to Human and Mouse B7 Immunoglobulin Superfamily Members.** Biolayer interferometry analysis of MIL33B binding to B7-H3 homologues and common immune checkpoint proteins demonstrated no significant affinity, confirming MIL33B specificity for multi-species B7-H3.

*Protein*	*KD(M)*	*KD Error*	*Full R^2*
**Human PD-L1**	> 1.6E-6	N/A	0
**Mouse PD-L1**	> 1.6E-6	1.15E-07	0
**Human PD-1**	> 1.6E-6	N/A	0
**Mouse PD-1**	> 1.6E-6	N/A	0
**Human PD-L2**	> 1.6E-6	1.24E-08	0
**Mouse PD-L2**	> 1.6E-6	3.77E-08	0
**Human B7-H2**	> 1.6E-6	4.52E-09	0
**Mouse B7-H2**	> 1.6E-6	1.0E-12	0
**Human B7-H4**	> 1.6E-6	4.19E-09	0
**Mouse B7-H4**	> 1.6E-6	2.21E-11	0

## Data Availability

All source data is contained within the text and Supplemental files or Tables.

## Abbreviations

RLT: Radioligand Therapy
EBRT: External Beam Radiation Therapy
ROS: Reactive Oxygen Species
TIME: Tumor Immune Microenvironment
PET-CT: Positron Emission Tomography (PET) - Computed Tomography (CT)
FOV: Field of View
mIF: Multiplexed Immunofluorescence
UMAP: Uniform Manifold Approximation Projection
Radio-SEC-HPLC: Radio-Size Exclusion Chromatography-High Pressure Liquid Chromatography
Bmax: Maximum Specific Binding
